# Who needs registered reports?

**DOI:** 10.1186/s12915-017-0394-2

**Published:** 2017-06-15

**Authors:** Miranda Robertson

**Affiliations:** BMC Biology, 236 Gray’s Inn Road, London, WC1X 8HB UK

## Abstract

Registered Reports, an article format initiated to help promote transparency and reproducibility in the preclinical and social sciences, are spreading into the biological literature. The format is now offered by *BMC Biology*, in a spirit of experiment.

## Dissenting views

On the possibly spurious grounds that an editorial is slightly less ephemeral than a blog, we are publishing an editorial to follow up our blog announcement [1] earlier this month that *BMC Biology* has added Registered Reports to the article formats it will consider for publication.

Since the announcement, we have collected the views of our Editorial Board on the format. They were – as always – interesting and instructive, and – as predicted – spread broadly across a spectrum from disapproval and deep scepticism to energetic enthusiasm. Their views, all of them valid, are represented in Q&A format below as a quick guide to what we think we’re doing.

Many of the questions reflect issues that have arisen before in the history of Registered Reports, and I have borrowed liberally from the extremely useful FAQs [2] on the Center for Open Science website (happily published under the Creative Commons License) in suggesting answers, as well as shamelessly quoting, with modification but without attribution, some of the remarks that came from the Board.

We will begin at the beginning with…

## What is a registered report?

A Registered Report – briefly – is an article format in which the rationale for a study and the proposed methodology (including statistical tests) are submitted for peer review before the data are collected. If the referees are satisfied that the question the study seeks to answer is well framed, and the proposed methodology appropriate to answer it, the Report is then accepted in principle irrespective of the outcome once the data are collected.

## Any old question provided that it’s well framed?

No. For *BMC Biology* the question must be of sufficient interest and/or importance to justify publication of the answer in a journal addressed to a broad readership.

## What if the result is negative?

A very important part of the reasoning behind Registered Reports is that if a question is important enough, the answer is important whether it’s yes or no. There is growing recognition that publication of negative results is important, and Registered Reports guarantee an author that if an experiment is well done, the result will still be published whichever way it goes.

## Why would anyone want to take the risk that they might be scooped by an unscrupulous reviewer?

This is a common concern, but the risk is not (realistically) very great, besides which the submission date of the Report will clearly precede that of any paper submitted by one of the reviewers later. And don’t forget, publication is guaranteed, whether or not someone else has arrived at the same result in the meantime.

## When you say publication is guaranteed, do you mean without further refereeing?

No. The final paper containing the data will be sent back to the referees. (The original submission is Stage 1; the final submission with data is Stage 2.) They will judge whether the methodology approved at Stage 1 has been followed, and valid conclusions have been drawn from the results. They may also be invoked earlier, if authors find they need, once they start experimenting, to adjust the original methodology.

## Isn’t it unrealistic to expect that much work from the referees?

It is astonishing how academic scientists will invest precious time in evaluating others’ work and making constructive suggestions on how it might be done better. Reviewing Registered Reports will (often) be more work than reviewing other sorts of paper, but it’s clearly possible to find referees who are willing – other journals already do, and we have some volunteers from among our Editorial Board.

## How could this format work for basic biology, where the course of the research is driven by the results as they are generated?

To the extent that different possible results can be foreseen, they can be incorporated in the original plan as contingencies, with the different paths to be followed built into the description of the methodology. Stage 2 papers can also report the results of exploratory investigations suggested by the results as they came in, provided that any such investigations are clearly stated to be such.

The format is not completely inflexible, although it certainly will not suit all biological research – indeed most of it.

## What sort of research questions will it be suitable for?

It was originally introduced for preclinical and psychological research, where the questions were of the kind ‘Does this treatment shrink this type of solid tumor?’ or ‘Is this EEG pattern a correlate of verbal processing?’ Questions in ecology, ethology and genomics are often similarly susceptible to such straightforward phrasing. Other biological experiments for which the format would be particularly useful would be

- Follow-ups to papers where an obvious question is raised by the results
- Experiments addressing issues on which controversy has arisen
- Similarly – experiments aimed at testing phenomena about which there is scepticism but that have not been definitively tested; or about which there are exaggerated claims

## How could you stop an author from submitting to a different journal at stage 2 if the results seemed very exciting?

We couldn’t. It’s not part of our remit to put authors in a strait jacket. In those circumstances, it’s likely that we’d publish Stage 1 as a Withdrawn Registration.

## You say there was a spectrum of views across your editorial board – were more for, or more against? or most neutral?

Of those who replied – so a self-selected sample – roughly half were for, about a third were sceptical, and the rest were against.

## Hm. Half of a self-selected sample, probably quite small. Do you think it’s enough?

Well; you may know what Mark Twain said about being in agreement with a majority. (I will refrain from topical reference to where majorities have recently got some of us.) The arguments for the format seem to us strong enough to justify the experiment. As with all experiments, there is no knowing whether it will work. We hope you will help us find out.
